# Management and Outcomes of Male Breast Cancer in Zaria, Nigeria

**DOI:** 10.1155/2012/845143

**Published:** 2012-09-06

**Authors:** Adamu Ahmed, Yahaya Ukwenya, Adamu Abdullahi, Iliyasu Muhammad

**Affiliations:** ^1^Department of Surgery, Ahmadu Bello University Teaching Hospital, Zaria, Nigeria; ^2^Department of Radiation Oncology, Ahmadu Bello University Teaching Hospital, 810001 Zaria, Nigeria

## Abstract

Male breast cancer is an uncommon disease accounting for only 1% of all breast cancers. We present the evaluation, treatment and outcome of male patients seen with breast cancer in our institution. Male patients that had histological diagnosis of breast cancer from 2001 to 2010 were retrospectively evaluated. After evaluation patients were treated with modified radical mastectomy. Combination chemotherapy was given to patients with positive axillary lymph nodes. Radiotherapy and hormonal therapy were also employed. There were 57 male patients with breast cancer which accounted for 9% of all breast cancers seen during the study period. Their mean age was 59 ± 2.3 years. The mean tumor diameter was 13 ± 2.5 cm. Fifty three (93%) patients presented with advanced disease including 15 with distant metastasis. Four patients with stage II disease were treated with modified radical mastectomy, chemotherapy and tamoxifen. Of the 30 patients with sage III disease that had modified radical mastectomy, complete axillary clearance and tumor free margins were achieved in 25. Overall 21 (36.8%) patients were tumor free at one year. Overall 5-year survival was 22.8%. In conclusion, male patients with breast cancer present with advanced disease which is associated with poor outcome of treatment.

## 1. Introduction

Male breast cancer (MBC) is an uncommon disease accounting for approximately 1% of all breast cancers diagnosed in the United States each year [1, 2].Of the 209,060 total cases of breast cancer expected in the United State in 2010, 1,970 (0.94%) will occur in men and 390 men are expected to die from the disease [3]. In contrast to the recent decline in incidence of female breast cancer (FBC), the incidence of MBC has been steadily increasing over the past three decades. The National Cancer Institute (NCI) Surveillance, Epidemiology, and End Results (SEER) Program noted increased incidence of MBC of 26% from 1973 to 1998 [4]. In the UK, current estimates indicate around 300 cases annually [5]. In contrast to western countries, the incidence of MBC in sub-Saharan Africa ranges from 1.3–15% [6–10]. 

In the past, incidence rates in Egypt were 12-times that of the United States but the current incidence rate (1.42%) is only slightly higher than the U.S. rate [11]. This has been attributed to the recent decline in Schistosoma parasitic infection and its associated liver fibrosis [11, 12]. In addition, it is estimated that approximately 10% of men with breast cancer have a genetic predisposition of which *BRCA2 *is the most clearly associated gene mutation [12, 13]. Associations have also been suggested with *BRCA 1*, *P53*, and *CHEK 2 *mutations [13, 14]. A recent study revealed that having a history of breast cancer in a brother is associated with a higher risk of breast cancer than having an affected sister suggesting that MBC has a higher genetic basis than FBC [13]. The Klinefelter syndrome, characterized by a 47-XXY karyotype, small testes, azospermia, and gynecomastia, has also been described as occurring in 3–7.5% of men with breast cancer [12]. Gynecomastia present in 6–38% of MBC patients has been described as a risk factor, although it is unclear whether gynecomastia is a risk factor for MBC or the risk factors for MBC are the same as those for gynecomastia [12, 14]. The contributions of several other variables including smoking, alcohol consumption, and exposure to electromagnetic radiation remain uncertain [14].

In sub-Saharan Africa patients with MBC present late with advanced disease and associated comorbidity [6, 7, 9]. These patients often seek nonorthodox treatment because of poor awareness, sociocultural or religious reasons. In addition, whilst surgical therapy is readily available other resources for breast cancer treatment may be lacking or very limited. These resulted in poor outcome of treatment [8, 9]. The rarity of MBC results in paucity of prospective randomized studies validating the efficacy of various treatment strategies for MBC. It has been suggested that in men, the behavior of breast carcinoma is similar to that in postmenopausal women [15]. The management of MBC, therefore, has been based on same principles of treatment as for female breast cancer. However, as more data on the tumor biology of male breast cancer emerge, it is becoming clear that it is a unique disease requiring its own treatment guidelines [16]. The objective of this study was to report the clinico-pathological features, treatment and outcome of patients with MBC seen in our institution over a 10 year period from 2001 to 2010.

## 2. Patients and Method

This retrospective study was carried out in the departments of surgery and radiation oncology Ahmadu Bello university teaching hospital Zaria, Nigeria. This hospital is an oncology center of excellence and one of the few institutions where functional radiotherapy facilities are available in Nigeria. All male patients that had histological diagnosis of breast carcinoma from January 2001 to December 2010 were included.

## 3. Patient Evaluation

Clinical diagnosis of breast cancer made from patient evaluation was confirmed with fine-needle aspiration cytology or incisional biopsy in ulcerated tumors. Staging of breast cancer was based on the International Union Against Cancer criteria [17]. Other investigations performed include full blood count, serum urea and electrolytes, and liver function test. Chest radiograph and abdominopelvic ultrasound were performed in appropriate cases.

## 4. Treatment Policy

Treatment was based on the tumor stage and patient's performance status. Patients with early or locally advanced disease were treated with modified radical mastectomy (MRM). Patients with metastatic disease were offered simple mastectomy as indicated. Cyclical combination chemotherapy was used if axillary lymph nodes were involved. This consisted of four weekly intravenous cyclophosphamide 1 g/m^2^, methotrexate 50 mg/m^2^ and 5-fluorouracil 600 mg/m^2^ (CMF). Radiotherapy was delivered using a cobalt-60 teletherapy beam. In radical radiotherapy, a dose of 50 Gy in 25 fractions over a 5-week period was used after mastectomy to the chest wall and supraclavicular lymph node regions. A similar dose was delivered to the axillary lymph nodes if they were not excised surgically. In palliative situations, a dose of 20–30 Gy in 10 fractions was given to either the breast or the metastatic lesions. Hormonal therapy using tamoxifen was offered to all patients at a daily dose of 20 mg for five years as estrogen receptors and progesterone receptors were not tested. Follow-up visits were scheduled every 1–3 months for 2 years and every six months thereafter. Followup blood and imaging studies were performed as required.

Data were entered into SPSS 17.0 (SPSS, Inc., Chicago, IL) statistical software. Frequencies, means, and standard deviations were determined. Overall survival (OS) was estimated with the Kaplan-Meier method. Multivariate analysis was performed using the Cox proportional hazards regression model to determine the independent association between each variable and survival. Variables that were considered in the multivariate analysis include patient age at diagnosis, disease stage, lymph node status, tumor grade, and histology. A *P*-value <0.05 was considered statistically significant.

## 5. Results

During the study period 635 patients with histological diagnosis of breast carcinoma were managed of which 57 (9.0%) were males. The characteristics of the male patients are shown in Table 1. Their mean age was 59 ± 2.3 years. Left and right breasts were affected in 31(54.4%) and 26(45.6%) patients respectively. Median duration of symptoms was 11 months. Twenty-eight (49.1%) patients presented primarily to our institution while 12 consulted traditional healers for 7 to 36 months. The most common symptoms were breast lump 45(79%), breast ulceration 25(43.9%) and nipple discharge (8.8%). The mean tumor diameter was 13 ± 2.5 cm. Other tumor characteristics are shown in Table 2. Fifteen patients presented with distant metastasis including three to the liver and five to the spine with spinal cord compression. 

## 6. Treatment

Patient's treatment is shown in Table 3. One patient with stage-II disease developed chest wall recurrence 19 months after surgery. Of the patients with advanced disease, five with distant metastasis and three with stage-III disease did not have any local surgery. Following surgery in patients with stage-III disease, complete axillary clearance and tumor-free margins were achieved in 25 patients. Seven patients required skin grafting postoperatively. Palliative radiotherapy given to nine patients resulted in good response in respect of control of pain, tumor size, and bleeding. The waiting times for radiotherapy ranged from 2–16 weeks. 

Overall, thirty-three (58%) patients completed six cycles of chemotherapy. Seven patients discontinued therapy after one to four cycles because of severe toxicity (2), progression of disease that made the patient unfit for chemotherapy (2) and financial constraints (3). Among the patients that completed chemotherapy three that did not have local surgery had regression of tumor lasting 9–13 months. Seventeen patients developed tumor recurrence between 13 and 42 months after chemotherapy; the most common site being the chest wall. All patients were given tamoxifen 20 mg daily. Twenty (35.1%) patients took tamoxifen for two or more years. 

At 6-month followup, 17(30%) patients had stopped coming to the clinic including five that died with metastatic disease. Thirty-eight (66.7%) patients were followed up for more than 1 year. Overall, 21 (36.8%) patients were tumor free at one year consisting of four (100%) with stage-II and 17 (51.5%) with stage-III disease. Two patients (50%) with stage-II disease were alive at 5 years. The 1-, 2-, and 5-year survival rates of patients with advanced disease were 60.4, 39.6, and 20.8%, respectively. The overall 5-year survival was 22.8%. The 5-year survival for patients with early disease was higher than for those with advanced disease (0.001). 

Univariate analysis indicated that patient age (0.01), stage of disease (0.001), extent of lymph node involvement (0.03), and histological type (0.03) were associated with survival. However, multiple regression analysis showed that only age at diagnosis (0.03), stage (0.001), lymph node status (0.001), and chemotherapy (0.001) were independent predictors of overall survival.

## 7. Discussion

There is a marked geographical difference in the world-wide incidence of MBC, with a higher incidence in the USA and the UK than in Finland and Japan [3]. In parts of Africa, the incidence of male breast cancer is relatively high. In a report from Zambia, 15% of breast cancer cases were male [7]. In the present study, males accounted for 9% of all breast cancers, similar to 9% reported from Zaria, 8.6% from Jos and 8% from Eastern Nigeria, respectively [8–10]. However, it is higher than other Nigerian studies including 1.47% from Nnewi and 2.5% from Benin [18, 19]. The prevalence of hepatitis-B virus infection in the West African subregion is 12.3% which together with high level of schistosomal infection result in liver cirrhosis that increase circulating estrogen levels and therefore, increased risk of MBC by stimulating breast tissue growth. In addition, androgen deficiency due to testicular diseases like mumps, undescended testes, testicular injury, and testicular torsion are not uncommon in our patients [20]. Finally, our institution being an oncology centre of excellence would attract patient's reference including those with MBC. These may partly explain the high prevalence of MBC in our patients. Prevention of chronic liver disease and early detection and treatment of testicular diseases will significantly reduce the risk of MBC in our patients.

**Figure 1:**
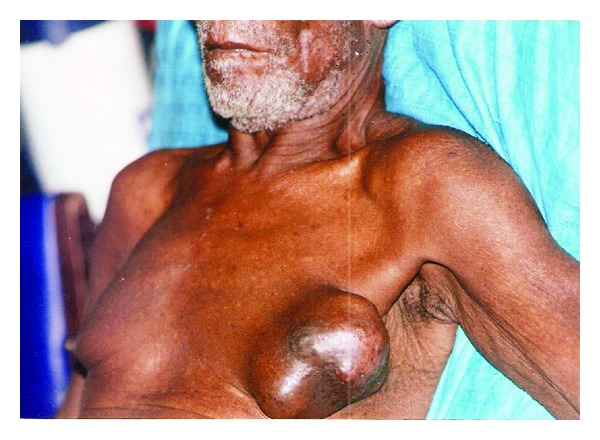
A hard central nontender mass in the left breast of a 64-year-old man.

The prevalence of male breast cancer increases with age, and it is rare before the age of 30 years. The mean age of 59 years in our patients agrees with other reports and is approximately 10 years older than in females with the disease [5, 11, 14]. In the present study, the youngest patient was 21 years old compared to a 12-year-old boy reported by Kidmas et al. [8]. In agreement with other studies, more tumors were located on the left compared to right breast [10, 19]. Collective reviews have shown predilection for the left side in a ratio of 1.07 : 1 [14]. Late presentation was common in our patients with a median duration of symptoms of 11 months, similar to 10–12 months reported from Nigeria but lower than 28 months from Morocco [19, 21]. In contrast, high-income countries reported mean duration of symptoms ranging from 1–8 months before diagnosis [14]. The typical presentation of breast cancer in men is a hard central nontender mass (Figure 1). The frequency of ulceration is higher in MBC compared to FBC because of the smaller size of their breast and advanced stage at presentation. Ulceration was present in 43.9% of our patients compared to 90% reported from Tanzania [6]. These patients may present with fungating and foul smelling tumors and presentation with tetanus has been reported [19]. Bilateral MBC has been reported in 1.9% of patients in high-income countries [22]. A recent study from Nigeria reported bilateral disease in 2.9% of patients [19]. However, bilateral breast cancer is rare in men and none of our patients presented with bilateral disease. In the present study, all patients presented with axillary lymphadenopathy similar to other reports from our subregion [6, 9, 23]. This is higher than 37.7%–55% reported from high-income countries [14, 24]. It has been suggested that because breast tumors in men are usually superficial and centrally located, these tumors have a ready access to dermal lymphatics, pectoral fascia and subareolar lymphatic channels [5]. Previous studies have shown a correlation between tumor size and risk of lymph node metastasis [21, 25]. In our patients the mean tumor size was 13 cm which is significantly larger than 2–3.5 cm reported from high-income countries [2, 14]. This is probably because men do not seek medical attention for breast masses as quickly as women. In 52.6% of our patients more than 4 lymph nodes were positive for metastasis, which partly accounts for the poor prognosis. In the National Cancer Database study which examined 4,755 men with breast cancer the frequency of stage III and Stage IV disease was 9.6% and 4.5%, respectively [25]. In the present study, the proportions of stage III and stage IV disease were 58% and 35%, respectively, similar to the findings of Ihekwaba from Ibadan and Hassan from Zaria about two decades earlier [9, 26]. Other studies from Africa reported stage IV disease in 43.8%–67.1% of their patients [19, 23, 27]. This presentation with advanced disease has been attributed to the small size of the male breast which leads to early invasion of skin and pectoral fascia. In addition, there is lack of awareness of MBC among patients and care givers leading to delayed presentation and delayed diagnosis, similar to our findings in FBC [28, 29]. Therefore, in addition to public enlightenment it is imperative for medical practitioners at various stages of health delivery system to send patients with suspected breast cancer to the appropriate treatment center as soon as possible. 

In our institution multimodality therapy utilizing surgery, radiation, and systemic therapy is the standard care for men with breast cancer. The surgical procedure performed on 60% of our patients was MRM. This agrees with 51–74% reported in other studies [8, 15, 21]. In our patients, MRM was carried out when complete extirpation of loco-regional disease was anticipated; otherwise simple mastectomy was performed as a toilet procedure. In high income countries, lumpectomy in combination with radiotherapy is performed in about 13% of patients [2, 14]. However, the use of lumpectomy is limited due to paucity of breast tissue in men which renders adequate tumor excision unlikely. In addition, presentation with large advanced tumors renders lumpectomy inappropriate, and so was not carried out in our patients. 

In high-income countries, radiotherapy is frequently employed in the treatment of MBC [30]. At the European Institute of Oncology in Milan, radiotherapy for MBC is recommended for tumors >1 cm or with more than one metastatic lymph node [30]. Other studies recommend radiotherapy for all patients with axillary nodal involvement [31, 32]. The rationale for postmastectomy radiotherapy is based on lack of breast tissue in male patients and the concern of inadequate surgical margin even in small tumors. In sub-Saharan Africa, radiotherapy is infrequently employed due to lack of facilities [14, 23]. In the present study, post-mastectomy radiotherapy was employed in 54.4% of patients and was associated with improved locoregional control and disease free survival. Local recurrence was observed in 21% of patients following radiotherapy which agrees with 3–29% reported in other studies [27, 29]. Recent studies have shown superior locoregional control, disease free survival (DFS) and OS with the use of radiotherapy after mastectomy for MBC [25, 30]. Radiotherapy was not employed in our patients with stage II disease. Previous studies indicate that adjuvant radiotherapy in early stage disease may not be necessary because the risk of local recurrence after adequate surgery is extremely low [2, 29].

Approximately 80–90% of cancers in men are ER positive while 65–90% are PR positive [14, 19]. Use of tamoxifen is associated with improved DFS and OS [33, 34]. A study of 135 MBC patients for a median followup of 14 years, demonstrated a clear benefit in terms of both recurrence and OS with the use of tamoxifen [34]. Assessment of estrogen and progesterone status of breast cancer is vital in determining hormonal manipulation in the treatment of breast cancer. This is not possible in most institutions in sub-Saharan Africa including our own thus, our patients had neither estrogen nor progesterone receptor determination. The potential benefit of tamoxifen is significantly greater than its minimal adverse effects and therefore all our patients were treated with tamoxifen. The efficacy of this approach has been demonstrated in other patients with advanced breast cancer and unknown estrogen receptor status [27, 30]. In the past, orchidectomy was the hormonal therapy employed in our institution [9, 20]. However, in the present study, patients were treated with tamoxifen similar to the findings in other recent studies [15, 33]. The use of tamoxifen avoids surgical morbidity resulting from ablative procedures, is more acceptable to men than orchidectomy, and is associated with good response [35]. 

Due to the substantial risk of recurrence and death from breast cancer, 58% of our patients received adjuvant chemotherapy. In the present study, CMF was used similar to the findings in other reports from sub-Saharan Africa [7, 10, 23]. A prospective study published by NCI on adjuvant chemotherapy with CMF in male patients with stage II breast cancer demonstrated a five-year actuarial survival of 82% with a median followup of 46 months indicating that chemotherapy is efficacious in node positive men [27]. In our patients as in others chemotherapy was associated with good clinical response including complete tumor regression, prevention of recurrence, and survival benefits [21, 25]. However, severe toxicities leading to discontinuation of chemotherapy were not uncommon.

In this series, the 5-year survival rate was 50% for stage-II disease and 20.8% for advanced disease. Other studies from Nigeria indicate overall survival ranging from 6 months to 3 years [10, 26, 27]. In the study from LAUTECH 57% of patients died from one week to seven months after diagnosis while an overall 3-year survival of 27.2% was reported from Enugu [10, 26]. The five-year OS in our patients was 22.8%. Similar poor survival rates were reported from other studies in low-income countries [7, 9, 23]. In high-income countries the five-year OS rates for all stages of breast cancer in men range from 36%–66%, and 10-year survival rates range from 17%–52% [31, 35]. In our patients, the factors that significantly affect prognosis were stage of disease, axillary lymph node status, and use of chemotherapy. Tumor stage and axillary nodal status have consistently been shown to be the most important independent predictors of overall survival [24, 33]. 

The limitations of this study include the retrospective nature of our data and the relatively small number of patients treated over a span of 10 years. In addition, followup was poor and irregular, and so it was difficult to know the exact time of recurrence or death. 

In conclusion, the proportion of males among all breast cancer cases is relatively high in our institution and most patients presented with advanced disease. Education of both patients and health providers is needed to increase awareness of MBC and ensure early presentation and prompt referral for early diagnosis and treatment. The treatment of men with breast cancer should be considered at a multidisciplinary forum and approach to management should still follow the established pattern for that of females. Modified radical mastectomy followed by external radiotherapy is the optimal treatment. As an adjuvant treatment, tamoxifen should be the first line approach in a majority of patients and chemotherapy is reserved for patients with poor prognostic factors. In patients with metastatic disease tamoxifen can be combined with chemotherapy for optimum palliation. However, these patients are elderly and have associated chronic diseases. Therefore, chemotherapy should be carefully designed if the desired improvement in quality of life is to be achieved. Palliative care of these patients may include adequate pain relief, reduction of tumor size or bleeding control with radiotherapy and control of offensive smell with appropriate dressing agent.

## Figures and Tables

**Table 1:** Characteristics of 57 male patients with breast cancer.

Characteristic	Number	%
Age (years)		
21–30	3	5.3
31–40	7	12.3
41–50	16	28.0
51–60	20	35.1
>60	11	19.3
Symptoms duration (months)		
1–3	8	14.1
4–6	13	22.8
7–12	19	33.3
>12	17	29.8
Family history of breast cancer (*n* = 41)		
Present	1	2.5
Absent	40	97.5
Tobacco smoking (*n* = 50)		
In the past	18	36.0
At present	7	14.0
Never	25	50.0
Alcohol drinking (*n* = 35)		
In the past	10	28.6
At present	4	11.4
Never	21	60.0

**Table 2:** Tumor characteristics in male patients with breast cancer.

Characteristics	Number	%
Tumor status		
T1	3	5.3
T2	11	19.3
T3	26	45.6
T4	17	29.8
Axillary lymph node status		
N1	9	15.8
N2	35	61.4
N3	13	22.8
Disease stage		
I	0	0
II	4	7.0
III	33	58.0
IV	20	35.0
Histological grade (*n* = 42)		
Well differentiated	15	35.7
Moderately differentiated	7	16.7
Poorly differentiated	20	47.6
Histological type (*n* = 42)		
Invasive ductal carcinoma	37	88.0
Papillary carcinoma	1	2.4
Invasive lobular	2	4.8
Invasive ductal and lobular	2	4.8

**Table 3:** Patient's surgery and adjuvant treatments according to TNM stage.

Stage II (*n* = 4)	Surgery	4 cases	MRM 4
	Radiotherapy		0
	Chemotherapy		4
	Hormonal therapy		4

Stage III (*n* = 33)	Surgery	30 cases	MRM 30
	Radiotherapy		22
	Chemotherapy		25
	Hormonal therapy		33

Stage IV (*n* = 20)	Surgery	15 cases	MRM 5
			SM 10
	Radiotherapy		9
	Chemotherapy		11
	Hormonal therapy		20

Modified radical mastectomy (MRM); Simple mastectomy (SM).
